# Chemotherapy-Related Amenorrhea and Quality of Life Among Premenopausal Women With Breast Cancer

**DOI:** 10.1001/jamanetworkopen.2023.43910

**Published:** 2023-11-16

**Authors:** Rayan Kabirian, Maria Alice Franzoi, Julie Havas, Charles Coutant, Olivier Tredan, Christelle Levy, Paul Cottu, Asma Dhaini Mérimèche, Sophie Guillermet, Jean-Marc Ferrero, Sylvie Giacchetti, Thierry Petit, Florence Dalenc, Philippe Rouanet, Sibille Everhard, Anne-Laure Martin, Barbara Pistilli, Matteo Lambertini, Ines Vaz-Luis, Antonio Di Meglio

**Affiliations:** 1 Department of Medical Oncology, Gustave Roussy, Villejuif, France; 2 Sorbonne Université, Paris, France; 3 INSERM Unit 981, Molecular Predictors and New Targets in Oncology, Gustave Roussy, Villejuif, France; 4 Centre Georges François Leclerc, Dijon, France; 5 Centre Léon Bérard, Lyon, France; 6 Centre François Baclesse, Caen, France; 7 Institut Curie, Paris, France; 8 Institut de Cancérologie de Lorraine–Alexis Vautrin, Vandœuvre-lès-Nancy, France; 9 Now with Centre Hospitalier de Lunéville-Ghemm, Pôle Mère-Enfant, Lunéville, France; 10 Centre Eugene-Marquis, Rennes, France; 11 Centre Antoine Lacassagne, Nice, France; 12 Hopital Saint Louis, Paris, France; 13 Centre Paul Strauss Centre de Lutte Contre le Cancer, Strasbourg, France; 14 Institut Claudius-Regaud, IUCT-Oncopole, Toulouse, France; 15 CRLC Val d’Aurelle, Montpellier, France; 16 Unicancer, Paris, France; 17 Department of Internal Medicine and Medical Specialties, School of Medicine University of Genova, Genova, Italy; 18 Department of Medical Oncology, UO Clinica di Oncologia Medica, IRCCS Ospedale Policlinico San Martino, Genova, Italy; 19 Département Interdisciplinaire d’Organisation des Parcours Patients, Gustave Roussy, Villejuif, France

## Abstract

**Importance:**

Younger survivors of breast cancer frequently report more treatment-related symptoms, mostly related to the menopausal transition.

**Objective:**

To assess factors associated with chemotherapy-related amenorrhea (CRA) and to evaluate its association with long-term quality of life (QOL).

**Design, Setting, and Participants:**

The prospective, longitudinal Cancer Toxicities Study, a multicenter French cohort study, includes women with a diagnosis of stage I to III breast cancer and collects data approximately yearly after diagnosis. The current study reports outcomes up to 4 years after diagnosis for participants enrolled from 2012 to 2017. Participants included premenopausal women younger than 50 years treated with chemotherapy and not receiving adjuvant ovarian function suppression. Data analysis was performed from September 2021 to June 2023.

**Exposures:**

Clinical, socioeconomic, tumor, and treatment characteristics assessed at diagnosis (for the analysis of factors associated with CRA) and persistent CRA (for the QOL analysis).

**Main Outcomes and Measures:**

The main outcome of interest was CRA at year 1 (Y1), year 2 (Y2), and year 4 (Y4) after diagnosis. Generalized estimating equations assessed associations of exposure variables with CRA. In the QOL analysis, QOL at Y4 (assessed with the European Organization for Research and Treatment of Cancer Quality of Life Questionnaires C30 and BR23) was the outcome of interest. Multivariable random-effect mixed models assessed the association of persistent CRA (ie, never recovering menses after treatment) with QOL.

**Results:**

Among 1636 women, the mean (SD) age at diagnosis was 42.2 (5.6) years. Overall, 1242 of 1497 women (83.0%) reported CRA at Y1, 959 of 1323 women (72.5%) reported it at Y2, and 599 of 906 women (66.1%) reported it at Y4. Older age vs 18 to 34 years (adjusted odds ratio [OR] for 35 to 39 years, 1.84 [95% CI, 1.32 to 2.56]; adjusted OR for 40 to 44 years, 5.90 [95% CI, 4.23 to 8.24]; and adjusted OR for ≥45 years, 21.29 [95% CI, 14.34 to 31.61]) and receipt of adjuvant tamoxifen (adjusted OR, 1.97 [95% CI, 1.53 to 2.53]) were associated with higher likelihood of CRA. In the QOL analysis, 416 of 729 women (57.1%) had persistent CRA. However, late menses recovery among women aged 18 to 34 years with no menses at Y2 were reported by 11 of 21 women (52.4%) between Y2 and Y4. Persistent CRA was associated with worse insomnia (mean difference vs recovery at any time, 9.9 points [95% CI, 3.2 to 16.5 points]; *P* = .004), systemic therapy–related adverse effects (mean difference, 3.0 points [95% CI, 0.2 to 5.8 points]; *P* = .04), and sexual functioning (mean difference, −9.2 points [95% CI, −14.3 to −4.1 points]; *P* < .001) at Y4.

**Conclusions and Relevance:**

In this cohort study of premenopausal women with breast cancer, persistent CRA was common, although some women recovered menses late, and was associated with worse long-term QOL. This study can help inform risk communication, personalized counseling, and early supportive care referrals for such patients.

## Key Points

**Question:**

Which factors are associated with chemotherapy-related amenorrhea, and what is its association with quality of life among breast cancer survivors?

**Findings:**

In this cohort study of 1636 premenopausal women, 416 of 729 women (57.1%) included in the quality of life analysis reported persistent amenorrhea after chemotherapy, although late recovery patterns were observed. Amenorrhea was more frequent among older women and those receiving adjuvant tamoxifen and was significantly associated with worse insomnia, systemic therapy–related adverse effects, and sexual dysfunction.

**Meaning:**

These findings can facilitate the treatment of premenopausal women with early breast cancer undergoing chemotherapy and experiencing chemotherapy-related amenorrhea, by informing risk communication, personalized counseling, and early supportive care referrals.

## Teaser

This cohort study assesses factors associated with chemotherapy-related amenorrhea and evaluates its association with long-term quality of life among premenopausal women with breast cancer.

## Introduction

Breast cancer is the most commonly diagnosed tumor in women, and approximately 20% of women with breast cancer are younger than 50 years at diagnosis.^1^ Compared with older women with breast cancer, younger survivors have higher risk of cancer-related symptoms and quality of life (QOL) deterioration^2,3^ and report worse depressive symptoms, higher level of stress, more weight gain, and greater difficulty managing the adverse effects of therapy.^4^ In particular, treatment-related symptoms linked to the menopausal transition (ie, vasomotor symptoms and sexual problems) represent an important source of distress during and after treatment, highlighting a need to monitor and address survivorship-related problems that are specific to this population.^5,6^

Chemotherapy-related amenorrhea (CRA) is among the possible adverse effects induced by treatment for breast cancer.^7,8^ There is no consensus definition of CRA across studies. According to the International Consensus Guidelines for Breast Cancer in Young Women,^9^ CRA can be empirically defined as absence of menses for at least 2 years, a persistent postmenopausal profile, and vaginal ultrasonography indicating that the ovaries are no longer functioning. Previous studies^10,11,12^ suggested that the prevalence of CRA ranges widely, from 11% to 96%, among women who are premenopausal at diagnosis of breast cancer and that it is associated with a number of clinical factors, including younger age at menarche, older age at breast cancer diagnosis, and lower body mass index, as well as treatment-related factors.^9,11,13,14,15^ Rates of CRA also may vary according to type of chemotherapy regimen^16^ and duration.^14^ Although some retrospective studies^13,17,18,19^ did not show a significant difference in the incidence of CRA with the addition of taxanes to an anthracycline-based regimen, some more recent and larger prospective cohort studies suggested increased rates of CRA among women receiving combination of a taxane and an anthracycline,^11,20^ irrespective of type of taxane (ie, docetaxel or paclitaxel).^21^ CRA may also have downstream outcomes on QOL. CRA is indeed often accompanied by symptoms associated with premature menopause and diminished ovarian reserve, including hot flashes, night sweats, vaginal dryness, concerns about infertility, and decreased sexual interest and enjoyment.^4,22^

There are still unanswered questions about CRA. There is a lack of longitudinal data regarding long-term trajectories of CRA and mid-term to long-term recovery patterns of menses, making it difficult for clinicians to give accurate estimations of the likelihood of recovery over time according to age range at diagnosis of breast cancer. Studies looking at treatment-related determinants of CRA in the era of currently standard combination chemotherapies including an anthracycline and a taxane or comparing different taxanes are also lacking. In addition, the association of persistent CRA with long-term patient-reported QOL has not been comprehensively explored. Using a large cohort with longitudinal patient characteristics and patient-reported outcomes, the present study aimed to (1) assess the prevalence of CRA and associated factors among women receiving standard anthracycline-based or taxane-based chemotherapy or a combination of both an anthracycline and a taxane and (2) evaluate the association of CRA with long-term QOL up to 4 years after breast cancer diagnosis.

## Methods

### Data Source

This study used data from CANTO (Cancer Toxicities Study), a French prospective, longitudinal, multicenter cohort study of women with stage I to III breast cancer.^23^ Briefly, patients are assessed at the time of breast cancer diagnosis (ie, before the start of any treatment), and then regular follow-up evaluations occur annually for up to 5 years. For the present analysis, relevant data were available at diagnosis and then at year 1 (Y1), year 2 (Y2), and year 4 (Y4) after diagnosis. A comprehensive assessment of clinical (including gynecological history, comorbidities, and behavioral factors), socioeconomic, tumor, and treatment data are available, as well as patient-reported outcomes assessing physical, psychological, and social domains of QOL. Patients who experience breast cancer recurrence (other than local), second cancers, or death exit the CANTO study at the time of the event. Enrollment covered 2012 to 2017. This study was approved by the French ethics committee and health authority. All patients provided written informed consent. All data were already available at the time of analysis, and no additional collection was performed. The study followed the Strengthening the Reporting of Observational Studies in Epidemiology (STROBE) reporting guidelines for cohort studies.^24^

### Study Cohort

We included women younger than 50 years^25^ with known premenopausal status at diagnosis, who received adjuvant and/or neoadjuvant chemotherapy. Women who (1) received ovarian function suppression using luteinizing hormone–releasing hormone agonists as part of adjuvant endocrine therapy, (2) underwent hysterectomy or oophorectomy either before or after diagnosis, and (3) had missing information about posttreatment menses status during all follow-up visits (Y1 and Y2 and Y4 after diagnosis) were excluded from the analysis. For the QOL analysis, women who had posttreatment menses data at all follow-up visit (Y1, Y2, and Y4 after diagnosis) were included (eFigure in Supplement 1).

### Variables of Interest

#### Primary Analysis

For the primary analysis, the outcome of interest was CRA, based on patient-reported menses status, dichotomized as the presence vs absence of menses. Exposure variables (all assessed at diagnosis) included age, comorbidities, health behaviors, sociodemographic characteristics, and tumor and treatment characteristics listed in Table 1.

**Table 1.** Patient and Tumor Characteristics at Baseline

Characteristic	Patients, No. (%) (N = 1636)^a^
Age at menarche, y	
<13	710 (45.2)
≥13	860 (54.8)
Missing	66
Age at diagnosis, mean (SD), y	42.2 (5.6)
Missing	0
Age group at diagnosis, y	
18-34	201 (12.3)
35-39	307 (18.8)
40-44	515 (31.5)
45-50	613 (37.5)
Missing	0
Presence of hot flashes at diagnosis	
Yes	210 (13.5)
No	1344 (86.5)
Missing	82
Charlson Comorbidity Index score	
0	1371 (90.4)
≥1	146 (9.6)
Missing	119
Body mass index^b^	
Normal (18.5 to <25)	974 (59.9)
Overweight or obese (≥25)	578 (35.5)
Underweight (<18.5)	74 (4.5)
Missing	10
Physical activity	
Insufficiently active (<10 metabolic equivalent of task h/wk)	642 (42.3)
Sufficiently active (≥10 metabolic equivalent of task h/wk)	876 (57.7)
Missing	118
Smoking status	
Current smoker	408 (25.4)
Former smoker	346 (21.5)
Never smoker	855 (53.1)
Missing	27
Daily alcohol consumption	
Yes	147 (9.3)
No	1435 (90.7)
Missing	54
Marital status	
Couple	1308 (85.9)
Single	215 (14.1)
Missing	113
Highest degree	
Primary or high school	645 (42.7)
College or higher	866 (57.3)
Missing	125
Monthly household income, €	
<1500	183 (12.2)
≥1500 to <3000	571 (37.9)
≥3000	751 (49.9)
Missing	131
Number of children^c^	
0	246 (15.0)
≥1	1390 (85.0)
Missing	0
Breast cancer stage	
I	398 (24.8)
II	921 (57.4)
III	286 (17.8)
Missing	31
Breast cancer histologic profile	
Ductal	1383 (84.8)
Others	247 (15.2)
Missing	6
Surgery	
Total mastectomy	683 (41.7)
Lumpectomy	953 (58.3)
Missing	0
Axillary surgery	
Dissection	976 (59.7)
None or sentinel node	660 (40.3)
Missing	0
Radiotherapy	
Yes	1544 (94.4)
No	91 (5.6)
Missing	1
Regimen of chemotherapy received^d^^,^^e^	
Anthracycline-based	57 (3.5)
Taxane-based	83 (5.1)
Combination of anthracyclines and taxanes	1496 (91.4)
Missing	0
Endocrine therapy^f^	
Yes	1228 (75.1)
No	408 (24.9)
Missing	0
Trastuzumab	
Yes	396 (24.2)
No	1240 (75.8)
Missing	0

^a^ Missing data were not included in calculations of percentages.

^b^ Body mass index is calculated as weight in kilograms divided by height in meters squared.

^c^ Women with missing data (n = 185) were considered without children. Association of this variable with chemotherapy-related amenorrhea was not tested in subsequent models.

^d^ Overall, 1571 of 1636 women (96.0%) received cyclophosphamide. The median number of chemotherapy cycles received was 4 in the taxane-based chemotherapy group, 5 in the anthracycline-based chemotherapy group, and 6 in the combination chemotherapy group.

^e^ Overall, 1238 of 1636 women (75.7%) received docetaxel, 192 of 1636 (11.7%) received paclitaxel, and 70 of 1636 (4.3%) received a sequence of the 2 agents.

^f^ All patients in this analysis were prescribed adjuvant tamoxifen as adjuvant endocrine therapy. Women not receiving any endocrine therapy either had hormone receptor–negative breast cancer or they refused or had contraindications to adjuvant tamoxifen.

#### QOL Analysis

For the secondary analyses, we focused on patient-reported outcomes assessed longitudinally at diagnosis (baseline), Y1, Y2, and Y4 (primary outcome) using the validated European Organization for Research and Treatment of Cancer (EORTC) Quality of Life Questionnaire (QLQ)–C30^26,27,28^ and QLQ-BR23. Worse QOL is reflected by a lower score for functional domains and by a higher score for symptom domains (score range, 0-100). Overall QOL was evaluated by the QLQ-C30 summary score (ie, obtained using the mean of 13 QLQ-C30 domains, excluding global health and financial impact, and scored as a functional domain).^29^

The exposure variable was CRA, dichotomized as never recovery of menses vs recovery at any time after diagnosis (either by Y1 [early recovery], or Y2 [late recovery], or Y4 [very late recovery]). Variables previously listed as exposure variables for the primary analysis served as covariates in the QOL analysis.

### Statistical Analysis

#### Primary Analysis

Descriptive statistics summarized cohort characteristics and rates of CRA over time, overall, and by age at breast cancer diagnosis. We then fit models to correlated responses by generalized estimating equations to explore the association of baseline exposure variables with the outcome of reporting CRA over time—that is, absence of menses at Y1, Y2, or Y4 after diagnosis. An exchangeable working correlation matrix structure was specified to account for within-patient correlations. The variance function for the binomial distribution and the logit-link function were used (binary response data). The specific contribution of chemotherapy type (ie, docetaxel vs paclitaxel) to the odds of CRA was investigated among patients receiving a taxane.

#### QOL Analysis

Descriptive statistics summarized menses status after diagnosis (never recovery of menses vs recovery by Y1, Y2, or Y4 after diagnosis). Mean estimates and changes in QOL outcomes (as a continuous variable) with respective 95% CIs were obtained using multivariable random-effect mixed models, adjusted by menses status group (vs recovery by Y1, Y2, or Y4 as a reference), time, menses status-by-time interactions, and covariates. Separate models were constructed for distinct QOL outcomes, each also including the respective QOL domain assessed at baseline.

Statistical analyses were performed with SAS statistical software version 9.4 (SAS Institute). All tests were based on previous evidence of association with CRA.^9,10,11,13,14,15,16,17,18,19,20^ In addition, we specifically tested the hypothesis that CRA is associated with worse QOL (ie, lower functional and higher symptom scores). Statistical significance was defined with a 2-sided *P* < .05. Data analysis was performed from September 2021 to June 2023.

## Results

### Cohort Characteristics

The analytic cohort included 1636 women for the primary analysis, of whom 1497 had menses data available at the Y1 evaluation, 1323 at Y2, and 906 at Y4 (eFigure in Supplement 1). The mean (SD) age at diagnosis was 42.2 (5.6) years (201 women [12.3%] were aged 18-34 years, 307 [18.8%] were aged 35-39 years, 515 [31.5%] were aged 40-44 years, and 613 [37.5%] were aged ≥45 years). Overall, 1496 women (91.4%) received a combination of anthracycline plus taxane, 57 (3.5%) received anthracycline-based chemotherapy without taxanes, and 83 (5.1%) received taxane-based chemotherapy without anthracyclines (overall, 1571 of 1636 women [96.0%] received cyclophosphamide in addition to either one of these regimens). Moreover, 1228 women (75.1%) received adjuvant endocrine therapy (all patients were prescribed tamoxifen), and 396 (24.2%) received trastuzumab. The median (IQR) time since chemotherapy completion was 7.4 (6.2-8.8) months at Y1, 19.8 (18.2-21.4) months at Y2, and 44.3 (42.4-46.0) months at Y4. Participant characteristics are presented in Table 1. Overall, 5 patients were pregnant at Y1, 9 were pregnant at Y2, and 16 were pregnant at Y4.

### Primary Analysis: Prevalence of CRA and Associated Factors

Overall, 1242 of 1497 women (83.0%) reported CRA at Y1, 959 of 1323 women (72.5%) reported CRA at Y2, and 599 of 906 women (66.1%) reported CRA at Y4. In the multivariable generalized estimating equation model, older age vs 18 to 34 years (adjusted odds ratio [OR] for 35-39 years, 1.84 [95% CI, 1.32-2.56]; adjusted OR for 40-44 years, 5.90 [95% CI, 4.23-8.24]; and adjusted OR for ≥45 years, 21.29 [95% CI, 14.34-31.61]), hot flashes at diagnosis (adjusted OR, 1.83 [95% CI, 1.17-2.86]), and receipt of adjuvant tamoxifen (adjusted OR, 1.97 [95% CI, 1.53-2.53]) were associated with higher likelihood of CRA. Among 1228 women taking tamoxifen, 1063 (86.6%) had CRA at Y1, 948 (77.2%) had CRA at Y2, and 844 (68.7%) had CRA at Y4. Patients receiving anthracycline-based chemotherapy vs anthracyclines-taxanes (adjusted OR, 0.35 [95% CI, 0.18-0.67]), those receiving taxane-based chemotherapy vs anthracyclines-taxanes (adjusted OR, 0.53 [95% CI, 0.31-0.89]), and those receiving trastuzumab (adjusted OR, 0.68 [95% CI, 0.52-0.89]) had lower likelihood of CRA. Full model results are shown in Table 2. There was no difference in the odds of CRA according to type of taxane received (adjusted OR for docetaxel vs paclitaxel, 1.00 [95% CI, 0.67-1.49]; 1430 patients). The full model is shown in eTable 1 in Supplement 1.

**Table 2.** Factors Associated With Chemotherapy-Related Amenorrhea in a Multivariable Generalized Estimating Equation Model

Characteristic	Adjusted OR (95% CI)^a^	*P* value
Age at menarche, <13 vs ≥13 y	0.96 (0.76-1.22)	.77
Age at diagnosis, y		
35-39 vs 18-34	1.84 (1.32-2.56)	<.001
40-44 vs 18-34	5.90 (4.23-8.24)	<.001
≥45 vs 18-34	21.29 (14.34-31.61)	<.001
Presence of hot flashes at diagnosis, yes vs no	1.83 (1.17-2.86)	.01
Charlson Comorbidity Index score, ≥1 vs 0	1.34 (0.89-2.02)	.16
Body mass index		
Overweight or obese vs normal weight	0.86 (0.67-1.11)	.25
Underweight vs normal weight	1.30 (0.70-2.40)	.40
Physical activity, sufficiently vs insufficiently active	0.98 (0.77-1.23)	.84
Smoking status		
Former smoker vs never smoker	0.97 (0.73-1.28)	.82
Current smoker vs never smoker	1.08 (0.81-1.43)	.60
Daily alcohol consumption, yes vs no	1.16 (0.76-1.77)	.50
Marital status, couple vs single	0.81 (0.58-1.15)	.24
Highest degree, college or higher vs primary or high school	0.91 (0.72-1.16)	.44
Breast cancer stage		
Stage II vs stage I	1.29 (0.96-1.74)	.08
Stage III vs stage I	1.20 (0.80-1.82)	.38
Breast cancer histologic profile, ductal vs others	0.78 (0.55-1.11)	.17
Surgery, total mastectomy vs lumpectomy	0.87 (0.67-1.12)	.28
Axillary surgery, dissection vs none or sentinel node	0.97 (0.73-1.29)	.83
Radiotherapy, yes vs no	0.74 (0.46-1.20)	.22
Chemotherapy regimen received		
Anthracycline-based vs anthracyclines-taxanes	0.35 (0.18-0.67)	.002
Taxane-based vs anthracyclines-taxanes	0.53 (0.31-0.89)	.02
Endocrine therapy, yes vs no	1.97 (1.53-2.53)	<.001
Trastuzumab, yes vs no	0.68 (0.52-0.89)	.005

Abbreviation: OR, odds ratio.

^a^ Adjusted for all factors in the table.

### QOL Analysis: Association of CRA With Long-Term QOL

Among 729 women with menses data available at all time points, cohort characteristics were consistent with the primary analysis (eTable 2 in Supplement 1). Characteristics of women not included in this analysis are available in eTables 3 and 4 in Supplement 1. Of note, 172 of 907 women not included had experienced recurrence, second cancer, or death and exited the CANTO study by Y4. A total of 416 patients (57.1%) reported having never recovered menses, whereas 313 (42.9%) had done so by the end of 4 years of follow-up. Specifically, across all age groups, 101 of 729 women (13.9%) had early menses recovery within Y1 after diagnosis, 133 of 628 women (21.2%) did so between Y1 and Y2, and 79 of 495 women (16.0%) recovered menses between Y2 and Y4.

**Figure 1.**
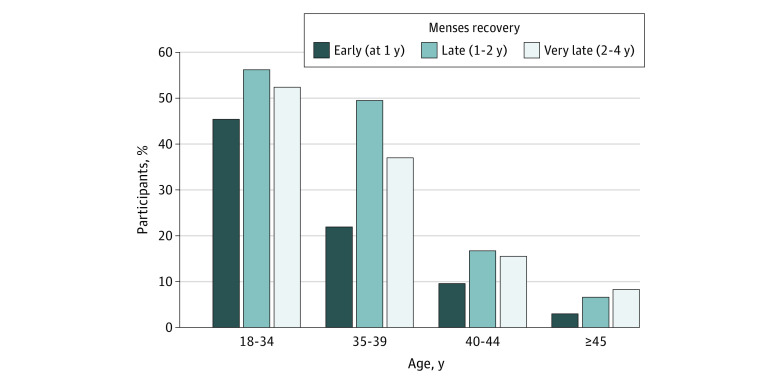
Menses Recovery Rate by Age Group Among 729 Women With Menses Status Available at All Time Points Overall, 313 patients (42.9%) reported menses recovery.

We then looked at the distribution of menses recovery rates by age group. Menses recovery by Y4 was reported by 78 of 88 women (88.6%) aged 18 to 34 years, 103 of 137 women (75.2%) aged 35 to 39 years, 87 of 239 women (36.4%) aged 40 to 44 years, and 45 of 265 women (17.0%) aged 45 years or older. As shown in Figure 1 and eTable 5 in Supplement 1, 11 of 21 women (52.4%) aged 18 to 34 years and 20 of 54 women (37.0%) aged 35 to 39 years who had amenorrhea at Y2 reported menses recovery by Y4. In the older age groups, 28 of 180 women (15.5%) in the 40 to 44 years group and 20 of 240 women (8.3%) in the 45 years or older group had menses recovery between Y2 and Y4 after diagnosis.

**Figure 2.**
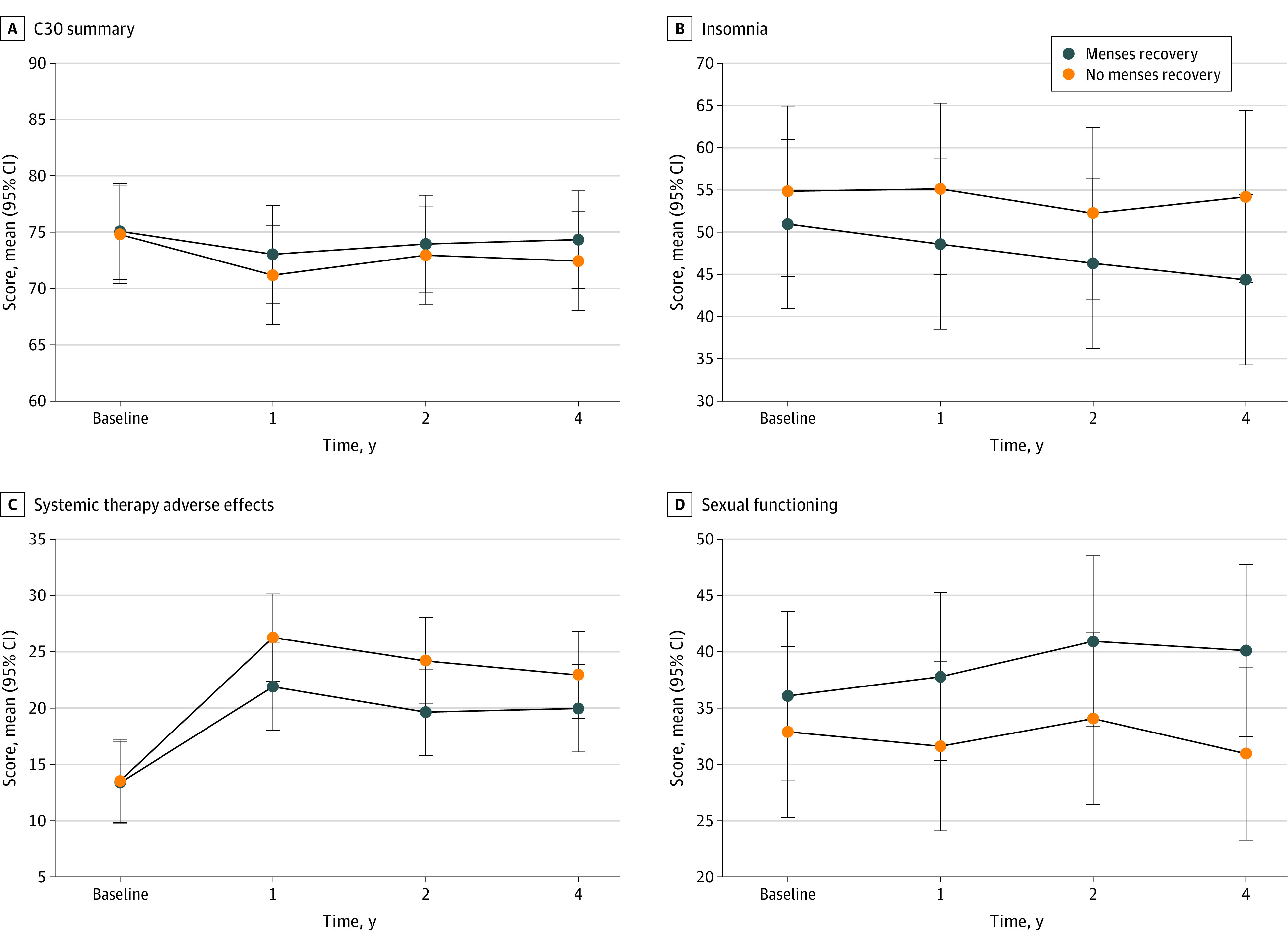
Longitudinal Evolution of Quality of Life From Diagnosis Through Year 1, Year 2, and Year 4 After Diagnosis Graphs show quality of life scores, including European Organization for Research and Treatment of Cancer Quality of Life Questionnaire C30 summary score (A), insomnia (B), systemic therapy adverse effects (C), and sexual functioning (D). Point estimates represent model-based mean estimates, with 95% CI error bars obtained using multivariable random-effect mixed models (adjusted by menses status group [vs recovery by year 1, 2, or 4 as a reference], time, menses status-by-time interactions, and covariates).

**Figure 3.**
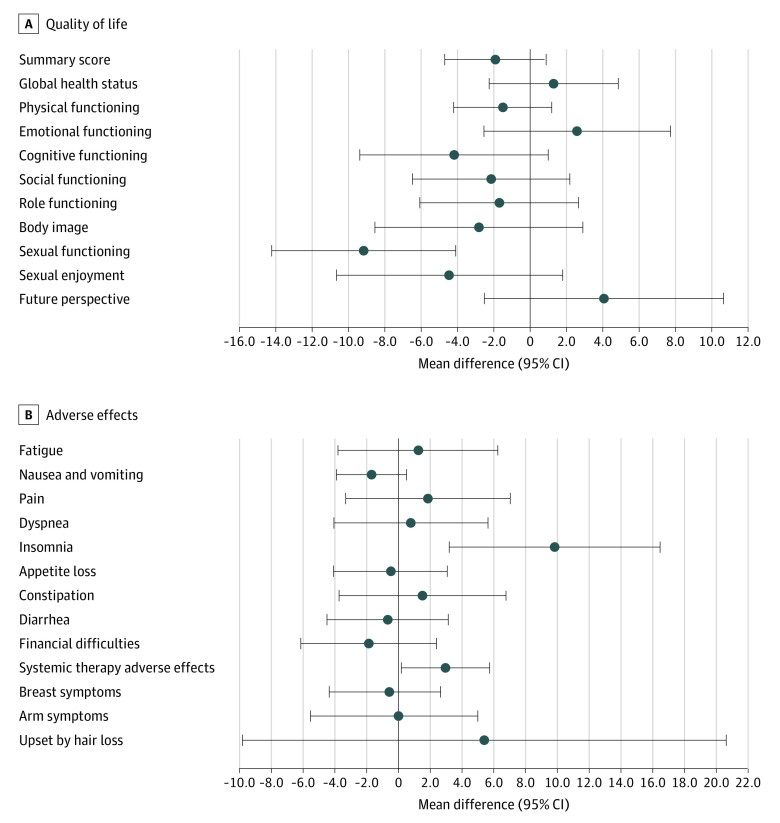
Mean Differences Between Quality of Life Scores at Year 4 Among Patients Reporting No Menses Recovery vs Recovery at Any Time After Diagnosis Point estimates represent model-based mean estimates with respective 95% CI error bars obtained using multivariable random-effect mixed models (adjusted by menses status group, time, menses status-by-time interactions, and covariates). A 95% CI not crossing the vertical zero line indicates a statistically significant between-group difference at year 4.

Compared with women who recovered menses by Y4, those who did not recover scored similarly for overall QOL (QLQ-C30 summary score mean difference, −1.9 points; 95% CI, −4.7 to 0.9 points; *P* = .18); however, they reported worse symptoms and more impaired functionality across some specific domains (Figure 2). In particular, never recovering menses was associated with more insomnia (mean difference, 9.9 points; 95% CI, 3.2 to 16.5 points; *P* = .004), more systemic therapy–related adverse effects (defined as having a number of chemotherapy-related adverse effects that include dry mouth, dysgeusia, hot flashes, headaches, and alopecia; mean difference, 3.0 points; 95% CI, 0.2 to 5.8 points; *P* = .04), and worse sexual functioning (mean difference, −9.2 points (95% CI, −14.3 to −4.1 points; *P* < .001) at Y4. Between-group estimate differences for the remaining domains at Y4 are reported in Figure 3.

## Discussion

This large, longitudinal cohort analysis of premenopausal women (mean age, 42.2 years) who received standard regimens of adjuvant and/or neoadjuvant chemotherapy for breast cancer helps answer several clinical questions about long-term trajectories of CRA and menses recovery rates by age and about factors associated with higher likelihood of CRA. It also provides a comprehensive longitudinal assessment of the association of menses recovery patterns with patient-reported QOL for over 4 years after breast cancer diagnosis.

Overall, we observed high rates of amenorrhea in this premenopausal cohort (83.0% at Y1, 72.5% at Y2, and 66.1% at Y4). Persistent amenorrhea was reported by 57.1% of women, with great age-related heterogeneity. Age represents the primary factor associated with the risk of premature ovarian failure and CRA.^12^ In the younger age groups of our analysis, many patients resumed menses by Y2, confirming prior results.^14,30,31^ Nevertheless, our longer-term data are also important and somehow reassuring. Even when a recovery is not observed by Y2, a large proportion of younger women seem to recover menses very late, between 2 and 4 years after diagnosis. These observations have several clinical implications. Before treatment inception, premenopausal women should be made aware of the risks associated with chemotherapy-related premature ovarian failure and persistent CRA and receive systematic oncofertility counseling.^32^ Among women younger than 39 years at the time of breast cancer diagnosis, 36% have not yet completed their family plans,^14^ and many of them may wish to have children after completion of treatment. Persistent or permanent CRA will necessarily cause delays or force these plans to change. In addition, in light of data showing possible late recoveries, contraceptive options should also be clearly discussed. Previous studies reported suboptimal contraceptive information for women not desiring pregnancy,^33^ with high rates of unintended pregnancies among breast cancer survivors.^34^ Dedicated gynecological counseling may help patients who have an inaccurate perception of infertility due to previous exposure to chemotherapy and long-term absence of menses.^33,35^

A late menses recovery pattern was also observed in older age groups in our analysis, with 8.3% of women older than 45 years reporting very late recovery between Y2 and Y4. This aspect can be challenging when it comes to the choice of the optimal adjuvant endocrine treatment. The absence of menses after completion of chemotherapy should not be used as a proxy for permanent transition to menopause, because it does not represent a reliable surrogate of gonadotoxicity.^9^ Adjuvant endocrine treatment choices should be based on a more thorough and comprehensive evaluation, combining absence of menses, assessments of circulating hormone levels, and gynecological ultrasonographic imaging.^9^

This study also helps identifying treatment-related determinants of CRA. Confirming some previous studies, we report higher rates of CRA among women treated with a combination of a taxane plus an anthracycline chemotherapy^11^ and/or with tamoxifen,^14^ and we found no difference in the odds of CRA according to type of taxane used, consistent with a previous study.^21^ The use of trastuzumab also did not seem to be associated with higher likelihood of CRA in CANTO, as previously shown.^36,37,38,39^

Furthermore, our study can inform personalized care pathways targeting patients at higher risk of QOL deterioration associated with a permanent menopausal transition. Women who never recovered menses had worse QOL, including more insomnia and other systemic therapy adverse effects at Y4 after diagnosis, similar to previous studies reporting difficulty sleeping and worse physical symptoms among women with CRA.^40^ Occurrence or exacerbation of menopausal symptoms caused by chemotherapy or endocrine therapy were suggested as a key precipitating factor for sleep problems and QOL deterioration.^2,3,41^ In particular, vasomotor symptoms have been positively correlated with sleep complaints in breast cancer survivors.^42,43^ In addition, we found substantial differences in patient-reported sexual function among women with persistent CRA compared with those who had recovered menses, as previously reported.^22^ Other analyses performed in the CANTO cohort also highlighted that sexual problems already may have been present before breast cancer treatment and seemed to either persist or worsen following primary therapy.^44^

### Limitations

This study is one the largest evaluating CRA prospectively in a contemporary cohort of premenopausal women with early breast cancer and also focusing on long-term QOL using internationally validated self-reported instruments, including a baseline point before treatment. However, some limitations should be acknowledged. We did not have access to the exact information on chemotherapy dose and scheduling. Therapeutic algorithms have recently evolved, including adding neoadjuvant carboplatin and immunotherapy for triple-negative breast cancer, which is common among younger women.^45^ Similarly, the impact of novel targeted agents, such as poly-ADP ribose polymerase inhibitors, pertuzumab, trastuzumab emtansine, or cyclin-dependent kinase 4 or 6 inhibitors, in the adjuvant setting could not be explored. Levels of antimullerian hormone or other markers of treatment-induced gonadotoxicity were not available, and CRA was self-reported. Ovarian function may resume while receiving tamoxifen even in the absence of menses, and this is difficult to determine without biochemical correlation.^46^ Adherence to endocrine therapy may also have influenced menses resumption (previous analyses among premenopausal women in CANTO found that 16.0%-36.2% of patients were nonadherent to tamoxifen).^47,48^ The study may not fully capture other potential confounding variables, including gender and ethnicity (the latter cannot be collected by French law). Generalizability of findings to patient populations other than ours (ie, premenopausal women aged <50 years, of whom most were aged 40-50 years, not receiving pharmacological ovarian function suppression) may be limited. As common in longitudinal studies, some dropout was observed, reducing the sample at later time points (particularly for the QOL analysis) and possibly introducing some selection bias toward patients experiencing a more favorable long-term disease course. However, the remaining cohort characteristics were balanced, and overall QOL scores were similar between the 2 groups. Given the hypothesis-driven approach of these analyses, we did not correct for multiple testing. Furthermore, some of the statistical differences in QOL that we found may correspond only to a small-to-moderate clinical between-group difference, based on previous studies using European Organization for Research and Treatment of Cancer QLQ-C30.^49^

## Conclusions

In conclusion, our findings can be used to inform clinical practice in several ways. Risk and duration of CRA, including potential late resumption of menses and its downstream implications for QOL, should be approached using a coordinated biopsychosocial model addressing multiple dimensions of physical, psychological, and social health. Proactive management of premenopausal women with early breast cancer undergoing chemotherapy should also include adapted strategies for risk communication, as well as personalized counseling and early supportive care referrals.

## Notes

Supplement 1. **eTable 1.** Factors Associated With Chemotherapy-Related Amenorrhea (CRA) in a Multivariable Generalized Estimating Equation Model Among Women Receiving Docetaxel or Paclitaxel (N=1430) **eTable 2.** Patient and Tumor Characteristics at Baseline Among Women With Menses Data Available at All Time Points and Included in the Quality of Life Analysis (N=729) **eTable 3.** Patient and Tumor Characteristics at Baseline Among Women Excluded From the Quality of Life Analysis (N=907) **eTable 4.** Description of Quality of Life Domain Scores Among Women With Menses Data Available at All Time Points and Included in the Quality of Life Analysis (n=729) and Those Excluded From the Quality of Life Analysis **eTable 5.** Menses Recovery Rate by Age Group Among Women With Menses Status Available at All Time Points (N=729) **eFigure.** Study Flowchart

Supplement 2.
Data Sharing Statement
