# Supplementary material for: Chemotherapy-Related Amenorrhea and Quality of Life Among Premenopausal Women With Breast Cancer
Source: JAMA Netw Open. 2023 Nov 16;6(11):e2343910. doi: 10.1001/jamanetworkopen.2023.43910 (PMC10654794; doi:10.1001/jamanetworkopen.2023.43910)
Supplement: Supplement 1. — eTable 1. Factors Associated With Chemotherapy-Related Amenorrhea (CRA) in a Multivariable Generalized Estimating Equation Model Among Women Receiving Docetaxel or Paclitaxel (N=1430) eTable 2. Patient and Tumor Characteristics at Baseline Among Women With Menses Data Available at All Time Points and Included in the Quality of Life Analysis (N=729) eTable 3. Patient and Tumor Characteristics at Baseline Among Women Excluded From the Quality of Life Analysis (N=907) eTable 4. Description of Quality of Life Domain Scores Among Women With Menses Data Available at All Time Points and Included in the Quality of Life Analysis (n=729) and Those Excluded From the Quality of Life Analysis eTable 5. Menses Recovery Rate by Age Group Among Women With Menses Status Available at All Time Points (N=729) eFigure. Study Flowchart [file jamanetwopen-e2343910-s001.pdf]

Supplemental Online Content

Kabirian R, Franzoi MA, Havas J, et al. Chemotherapy-related amenorrhea and quality of life among premenopausal women with breast cancer. *JAMA Netw Open*. 2023;6(11):e2343910. doi:10.1001/jamanetworkopen.2023.43910

eTable 1. Factors Associated With Chemotherapy-Related Amenorrhea (CRA) in a Multivariable Generalized Estimating Equation Model Among Women Receiving Docetaxel or Paclitaxel (N=1430)
eTable 2. Patient and Tumor Characteristics at Baseline Among Women With Menses Data Available at All Time Points and Included in the Quality of Life Analysis (N=729)
eTable 3. Patient and Tumor Characteristics at Baseline Among Women Excluded From the Quality of Life Analysis (N=907)
eTable 4. Description of Quality of Life Domain Scores Among Women With Menses Data Available at All Time Points and Included in the Quality of Life Analysis (n=729) and Those Excluded From the Quality of Life Analysis
eTable 5. Menses Recovery Rate by Age Group Among Women With Menses Status Available at All Time Points (N=729)
eFigure. Study Flowchart

This supplemental material has been provided by the authors to give readers additional information about their work.

eTable 1. Factors Associated With Chemotherapy-Related Amenorrhea (CRA) in a Multivariable Generalized Estimating Equation Model Among Women Receiving Docetaxel or Paclitaxel (N=1430)

Characteristic	Odds Ratio	Lower limit for Confidence interval	Upper limit for Confidence interval	*p-value*
**Age at menarche**				
<13 years old vs ≥13 years old	0.99	0.77	1.26	0.91
**Age at diagnosis**				
35-39 years old vs 18-34 years old	1.85	1.30	2.62	0.001
40-44 years old vs 18-34 years old	5.92	4.17	8.40	
≥45 years old vs 18-34 years old	19.35	12.75	29.37	
**Presence of hot flashes at diagnosis**				
Yes vs No	2.29	1.36	3.84	0.002
**Charlson comorbidity index**				
≥1 vs 0	1.22	0.81	1.83	0.34
**Body Mass Index**				
Overweight or obese vs Normal weight	0.83	0.64	1.08	0.16
Underweight vs Normal weight	1.29	0.67	2.50	0.45
**Physical Activity**				
Sufficiently vs Insufficiently active	0.99	0.77	1.27	0.96
**Smoking status**				
Former smoker vs Never smoker	0.97	0.72	1.30	0.84
Current smoker vs Never smoker	1.06	0.79	1.43	0.70
**Daily alcohol consumption**				
Yes vs No	1.13	0.74	1.74	0.57
**Marital status**				
Couple vs Single	0.76	0.52	1.10	0.15
**Highest degree**				
College or higher vs Primary or high school	0.87	0.67	1.13	0.30
**Breast Cancer stage**				
Stage II vs Stage I	1.38	1.00	1.90	0.049

© 2023 Kabirian R et al. *JAMA Network Open.*

Stage III vs Stage I	1.32	0.85	2.06	0.22
**Breast Cancer Histology**				
Ductal vs Others	0.81	0.55	1.20	0.30
**Surgery**				
Total mastectomy vs Lumpectomy	0.88	0.67	1.15	0.34
**Axillary surgery**				
Dissection vs None or sentinel node	1.00	0.74	1.35	0.99
**Radiotherapy**				
Yes vs No	0.71	0.43	1.17	0.18
**Type of taxane received**				
Docetaxel vs. paclitaxel	1.00	0.67	1.49	0.98
**Endocrine therapy**				
Yes vs No	1.93	1.48	2.53	<0.001
**Trastuzumab**				
Yes vs No	0.63	0.48	0.83	0.001
Adjusted for all factors in the table.				

© 2023 Kabirian R et al. *JAMA Network Open.*

eTable 2. Patient and Tumor Characteristics at Baseline Among Women With Menses Data Available at All Time Points and Included in the Quality of Life Analysis (N=729)

Characteristic	No[1] (%)
**Age at menarche:**	
< 13 years old	307 (43.7)
≥13 years old	396 (56.3)
Missing	26
**Age at diagnosis:**	
Mean (SD)	42.2 (5.4)
Missing	-
**Age at diagnosis:**	
18-34 years old	88 (12.1)
35-39 years old	137 (18.8)
40-44 years old	239 (32.8)
≥45 years old	265 (36.3)
Missing	-
**Presence of hot flashes at diagnosis:**	
Yes	83 (11.9)
No	612 (88.1)
Missing	34
**Charlson comobidity index:**	
0	616 (89.7)
≥1	71 (10.3)
Missing	42
**Body Mass Index**	
Normal (18.5-25 kg/m^2)	435 (59.7)
Overweight or Obese (≥25 kg/m^2)	256 (35.2)
Underweight (<18.5 kg/m^2)	37 (5.1)
Missing	1
**Physical Activity:**	
Insufficiently active	286 (41.0)
Sufficiently active	412 (59.0)
Missing	31
**Smoking status:**	
Current smoker	169 (23.6)
Former smoker	154 (21.5)
Non smoker	394 (54.9)
Missing	12
**Daily alcohol consumption:**	
Yes	57 (8.1)
No	650 (91.9)
Missing	22
**Marital status:**	
Couple	611 (86.9)
Single	92 (13.1)
Missing	26
**Highest degree:**	
Primary or high school	276 (39.5)
College or higher	422 (60.5)
Missing	31
**Income:**	

© 2023 Kabirian R et al. *JAMA Network Open.*

<1500 euros	79 (11.3)
≥1500 and <3000 euros	253 (36.3)
> 3000 euros	365 (52.4)
Missing	32
**Number of children[2]:**	
0	102 (14.0)
≥ 1	627 (86.0)
Missing	-
**Breast Cancer stage:**	
Stage I	208 (29.2)
Stage II	415 (58.2)
Stage III	90 (12.6)
Missing	16
**Breast Cancer Histology:**	
Ductal	615 (84.5)
Others	113 (15.5)
Missing	1
**Surgery:**	
Total mastectomy	285 (39.1)
Lumpectomy	444 (60.9)
Missing	-
**Axillary surgery:**	
Dissection	410 (56.2)
None or sentinel node	319 (43.8)
Missing	-
**Radiotherapy:**	
Yes	680 (93.4)
No	48 (6.6)
Missing	1
**Regimen of chemotherapy received:**	
Anthracycline-based	28 (3.8)
Taxane-based	40 (5.5)
Combination of anthracycline and taxane	661 (90.7)
Missing	-
**Endocrine therapy:**	
Yes	562 (77.1)
No	167 (22.9)
Missing	-
**Trastuzumab:**	
Yes	164 (22.5)
No	565 (77.5)
Missing	-
[1]No = Number	
[2]women with missing data (n=77) were considered without children.	

© 2023 Kabirian R et al. *JAMA Network Open.*

**eTable 3. Patient and Tumor Characteristics at Baseline Among Women Excluded From the Quality of Life Analysis (N=907)**	
**Characteristic**	**No[1] (%)**
**Age at menarche:**	
< 13 years old	403 (46.5)
≥13 years old	464 (53.5)
Missing	40
**Age at diagnosis:**	
Mean (SD)	42.2 (5.7)
Missing	-
**Age at diagnosis:**	
18-34 years old	113 (12.5)
35-39 years old	170 (18.7)
40-44 years old	276 (30.4)
≥45 years old	348 (38.4)
Missing	-
**Presence of hot flashes at diagnosis:**	
Yes	127 (14.8)
No	732 (85.2)
Missing	48
**Charlson comorbidity index:**	
0	755 (91.0)
≥1	75 (9.0)
Missing	77
**Body Mass Index**	
Normal (18.5-25 kg/m^2)	539 (60.0)
Overweight or Obese (≥25 kg/m^2)	322 (35.9)
Underweight (<18.5 kg/m^2)	37 (4.1)
Missing	9
**Physical Activity:**	
Insufficiently active	356 (43.4)
Sufficiently active	464 (56.6)
Missing	87
**Smoking status:**	
Current smoker	239 (26.8)
Former smoker	192 (21.5)
Non smoker	461 (51.7)
Missing	15
**Daily alcohol consumption:**	
Yes	90 (10.3)
No	785 (89.7)
Missing	32
**Marital status:**	
Couple	697 (85.0)
Single	123 (15.0)
Missing	87
**Highest degree:**	
Primary or high school	369 (45.4)
College or higher	444 (54.6)
Missing	94
**Income:**	
<1500 euros	104 (12.9)

© 2023 Kabirian R et al. *JAMA Network Open.*

≥1500 and <3000 euros	318 (39.4)
> 3000 euros	386 (47.8)
Missing	99
**Number of children[2]:**	
0	144 (15.9)
≥ 1	763 (84.1)
Missing	-
**Breast Cancer stage:**	
Stage I	190 (21.3)
Stage II	506 (56.7)
Stage III	196 (22.0)
Missing	15
**Breast Cancer Histology:**	
Ductal	768 (85.1)
Others	134 (14.9)
Missing	5
**Surgery:**	
Total mastectomy	398 (43.9)
Lumpectomy	509 (56.1)
Missing	-
**Axillary surgery:**	
Dissection	566 (62.4)
None or sentinel node	341 (37.6)
Missing	-
**Radiotherapy:**	
Yes	864 (95.3)
No	43 (4.7)
Missing	-
**Regimen of chemotherapy received:**	
Anthracycline-based	29 (3.2)
Taxane-based	43 (4.7)
Combination of anthracycline and taxane	835 (92.1)
Missing	-
**Endocrine therapy:**	
Yes	666 (73.4)
No	241 (26.6)
Missing	-
**Trastuzumab:**	
Yes	232 (25.6)
No	675 (74.4)
Missing	-

[1]No = Number

[2]women with missing data were considered without children. Association of this variable with chemotherapy-related amenorrhea was not tested in subsequent models.

© 2023 Kabirian R et al. *JAMA Network Open.*

eTable 4. Description of Quality of Life Domain Scores Among Women With Menses Data Available at All Time Points and Included in the Quality of Life Analysis (n=729) and Those Excluded From the Quality of Life Analysis

	**Patients included in the quality of life analysis (n=729)**				**Patients excluded from the quality of life analysis (n=907)**			
**Variable at Baseline**	**N**	**Mean score**	**Lower 95% CL for Mean**	**Upper 95% CL for Mean**	**N**	**Mean score**	**Lower 95% CL for Mean**	**Upper 95% CL for Mean**
Summary Score	686	80.29	79.30	81.28	803	80.76	79.84	81.67
Global health status	695	66.91	65.51	68.31	817	66.89	65.56	68.22
Physical Functioning	702	93.65	92.84	94.46	822	93.22	92.41	94.03
Emotional Functioning	700	58.55	56.71	60.39	820	59.93	58.23	61.62
Cognitive Functioning	699	78.09	76.28	79.90	821	79.01	77.40	80.62
Social Functioning	691	86.93	85.37	88.49	816	86.91	85.44	88.37
Role Functioning	701	83.31	81.56	85.06	822	82.87	81.21	84.53
Fatigue	702	34.91	33.02	36.80	820	35.01	33.23	36.78
Nausea and vomiting	702	6.05	5.01	7.09	821	5.40	4.53	6.27
Pain	702	18.19	16.60	19.77	823	19.60	18.00	21.20
Dyspnea	701	10.41	8.88	11.95	820	8.98	7.64	10.33
Insomnia	702	45.73	43.21	48.25	821	45.43	43.04	47.82
Appetite loss	700	21.81	19.76	23.86	820	20.45	18.56	22.34
Constipation	699	10.30	8.77	11.83	817	9.06	7.65	10.46
Diarrhea	700	9.33	7.92	10.74	819	9.28	7.96	10.60
Financial difficulties	683	9.76	8.11	11.41	808	8.91	7.51	10.31
Body image	692	86.34	84.77	87.91	806	84.94	83.47	86.41
Sexual functioning	689	35.90	33.97	37.82	799	35.23	33.34	37.13
Sexual enjoyment	484	66.67	64.21	69.13	538	67.97	65.69	70.24
Future perspective	692	40.46	38.07	42.85	804	41.50	39.27	43.73
Systemic therapy side effects	698	10.03	9.20	10.85	812	10.67	9.80	11.53
Breast symptoms	646	16.50	15.07	17.92	754	18.69	17.26	20.13
Arm symptoms	693	14.72	13.07	16.37	799	15.23	13.60	16.86
Upset by hair loss	92	25.36	18.86	31.86	103	33.98	26.97	40.99

© 2023 Kabirian R et al. *JAMA Network Open.*

Variable at Y1	N	Mean score	Lower 95% CL for Mean	Upper 95% CL for Mean	N	Mean score	Lower 95% CL for Mean	Upper 95% CL for Mean
Summary Score	674	77.07	75.95	78.20	743	77.34	76.21	78.47
Global health status	683	67.61	66.33	68.88	751	67.57	66.26	68.87
Physical Functioning	691	84.79	83.69	85.88	766	85.50	84.49	86.51
Emotional Functioning	692	68.02	66.13	69.91	766	69.17	67.38	70.97
Cognitive Functioning	692	71.70	69.75	73.65	766	73.06	71.19	74.93
Social Functioning	689	78.23	76.33	80.13	761	77.14	75.27	79.00
Role Functioning	692	75.87	73.97	77.77	766	76.57	74.77	78.37
Fatigue	692	40.95	39.04	42.86	766	40.64	38.78	42.51
Nausea and vomiting	692	5.54	4.48	6.60	765	6.47	5.34	7.61
Pain	692	30.47	28.58	32.36	766	30.46	28.46	32.46
Dyspnea	687	22.66	20.70	24.62	757	20.43	18.58	22.28
Insomnia	690	46.57	43.92	49.22	764	45.77	43.20	48.33
Appetite loss	689	9.00	7.51	10.48	765	8.93	7.49	10.37
Constipation	690	16.86	14.83	18.89	765	16.86	14.93	18.80
Diarrhea	692	8.62	7.20	10.04	761	10.64	9.04	12.25
Financial difficulties	686	20.75	18.42	23.08	753	22.40	20.18	24.62
Body image	687	60.16	57.81	62.52	758	58.62	56.31	60.92
Sexual functioning	679	34.71	32.90	36.52	745	34.92	33.06	36.78
Sexual enjoyment	505	58.22	55.83	60.60	518	59.27	56.93	61.60
Future perspective	687	44.59	42.21	46.97	755	46.49	44.15	48.83
Systemic therapy side effects	689	21.67	20.41	22.93	763	21.07	19.83	22.32
Breast symptoms	689	27.87	26.38	29.37	760	27.38	25.91	28.86
Arm symptoms	689	30.94	28.91	32.96	762	30.95	28.96	32.94
Upset by hair loss	94	64.54	56.23	72.84	108	67.59	60.35	74.84
**Variable at Y2**	**N**	**Mean score**	**Lower 95% CL for Mean**	**Upper 95% CL for Mean**	**N**	**Mean score**	**Lower 95% CL for Mean**	**Upper 95% CL for Mean**
Summary Score	658	78.64	77.54	79.74	590	78.94	77.81	80.07
Global health status	662	67.03	65.65	68.41	595	67.65	66.17	69.13

© 2023 Kabirian R et al. *JAMA Network Open.*

Physical Functioning	667	86.79	85.75	87.84	604	87.32	86.27	88.38
Emotional Functioning	667	66.96	65.02	68.90	603	68.11	66.07	70.14
Cognitive Functioning	667	70.59	68.53	72.65	603	72.66	70.62	74.71
Social Functioning	666	83.11	81.33	84.89	603	83.94	82.15	85.74
Role Functioning	667	81.58	79.86	83.31	603	81.76	79.90	83.61
Fatigue	667	39.34	37.46	41.22	602	39.36	37.34	41.38
Nausea and vomiting	667	5.17	4.18	6.17	602	5.48	4.37	6.60
Pain	667	27.79	25.86	29.71	602	28.52	26.45	30.58
Dyspnea	667	19.59	17.71	21.47	602	18.83	16.77	20.88
Insomnia	665	43.41	40.79	46.03	600	43.11	40.25	45.97
Appetite loss	666	8.06	6.66	9.46	602	7.59	6.14	9.03
Constipation	665	16.84	14.82	18.87	602	18.60	16.35	20.86
Diarrhea	665	7.57	6.17	8.96	599	8.35	6.76	9.93
Financial difficulties	667	13.59	11.56	15.62	599	11.91	9.88	13.94
Body image	655	69.08	66.73	71.43	596	67.15	64.62	69.69
Sexual functioning	653	37.77	35.78	39.77	588	37.05	34.93	39.16
Sexual enjoyment	485	62.75	60.24	65.26	415	61.53	58.82	64.23
Future perspective	653	53.55	51.04	56.06	594	54.60	51.92	57.28
Systemic therapy side effects	659	18.78	17.68	19.88	602	18.29	17.09	19.48
Breast symptoms	657	21.77	20.39	23.16	596	22.06	20.46	23.67
Arm symptoms	657	26.64	24.64	28.63	597	26.63	24.47	28.79
Upset by hair loss	124	47.31	40.02	54.60	111	44.14	36.96	51.33
**Variable at Y4**	**N**	**Mean score**	**Lower 95% CL for Mean**	**Upper 95% CL for Mean**	**N**	**Mean score**	**Lower 95% CL for Mean**	**Upper 95% CL for Mean**
Summary Score	551	78.80	77.61	80.00	271	80.39	78.60	82.19
Global health status	553	67.01	65.50	68.53	275	67.73	65.36	70.09
Physical Functioning	561	87.78	86.61	88.95	275	88.65	87.01	90.30
Emotional Functioning	560	66.98	64.88	69.08	276	69.76	66.74	72.78
Cognitive Functioning	560	72.44	70.29	74.59	276	73.19	70.06	76.32
Social Functioning	559	84.29	82.41	86.17	275	85.45	82.70	88.21

© 2023 Kabirian R et al. *JAMA Network Open.*

Role Functioning	560	83.30	81.47	85.14	275	83.15	80.32	85.98
Fatigue	561	39.85	37.80	41.90	275	35.68	32.50	38.86
Nausea and vomiting	561	4.81	3.83	5.80	276	5.43	3.59	7.27
Pain	561	27.63	25.45	29.81	276	27.78	24.44	31.12
Dyspnea	559	20.51	18.41	22.62	276	15.82	12.99	18.65
Insomnia	560	44.58	41.81	47.36	276	39.49	35.34	43.64
Appetite loss	560	7.50	6.03	8.97	276	9.30	6.65	11.95
Constipation	560	18.57	16.27	20.88	275	15.88	12.65	19.11
Diarrhea	558	8.96	7.37	10.55	274	6.93	4.72	9.15
Financial difficulties	557	8.26	6.50	10.02	275	7.52	5.10	9.93
Body image	554	72.81	70.25	75.37	274	73.08	69.39	76.77
Sexual functioning	551	35.30	33.12	37.48	273	35.16	31.94	38.39
Sexual enjoyment	392	61.48	58.67	64.29	182	61.36	57.12	65.59
Future perspective	551	58.86	56.17	61.55	274	60.34	56.31	64.37
Systemic therapy side effects	554	18.26	17.03	19.49	274	17.46	15.89	19.03
Breast symptoms	554	18.37	16.95	19.78	273	17.61	15.55	19.68
Arm symptoms	554	23.65	21.42	25.87	273	23.63	20.36	26.89
Upset by hair loss	152	42.76	36.45	49.08	79	40.93	32.82	49.03
N=number of patients ; CL = Confidence Limit								

© 2023 Kabirian R et al. *JAMA Network Open.*

eTable 5. Menses Recovery Rate by Age Group Among Women With Menses Status Available at All Time Points (N=729)
Overall, 42.9% patients (N=313/729) reported menses recovery.
*Denominator indicates total N. of patients assessed at each time point minus those who had recovered menses by the previous assessment.

	**Menses recovery at Y1 (Early recovery)**	**Menses recovery between Y1 and Y2 (Late recovery)**	**Menses recovery between Y2 and Y4 (Very late recovery)**	**Total percentage of menses recovery by Y4**
**18-34 years old**	45.4 (40/88)	56.2 (27/48*)	52.4 (11/21*)	88.6 (78/88)
**35-39 years old**	21.9 (30/137)	49.5 (53/107*)	37.0 (20/54*)	75.2 (103/137)
**40-44 years old**	9.6 (23/239)	16.7 (36/216*)	15.5 (28/180*)	36.4 (87/239)
**≥45 years old**	3.0 (8/265)	6.6 (17/257*)	8.3 (20/240*)	17.0 (45/265)

© 2023 Kabirian R et al. *JAMA Network Open.*

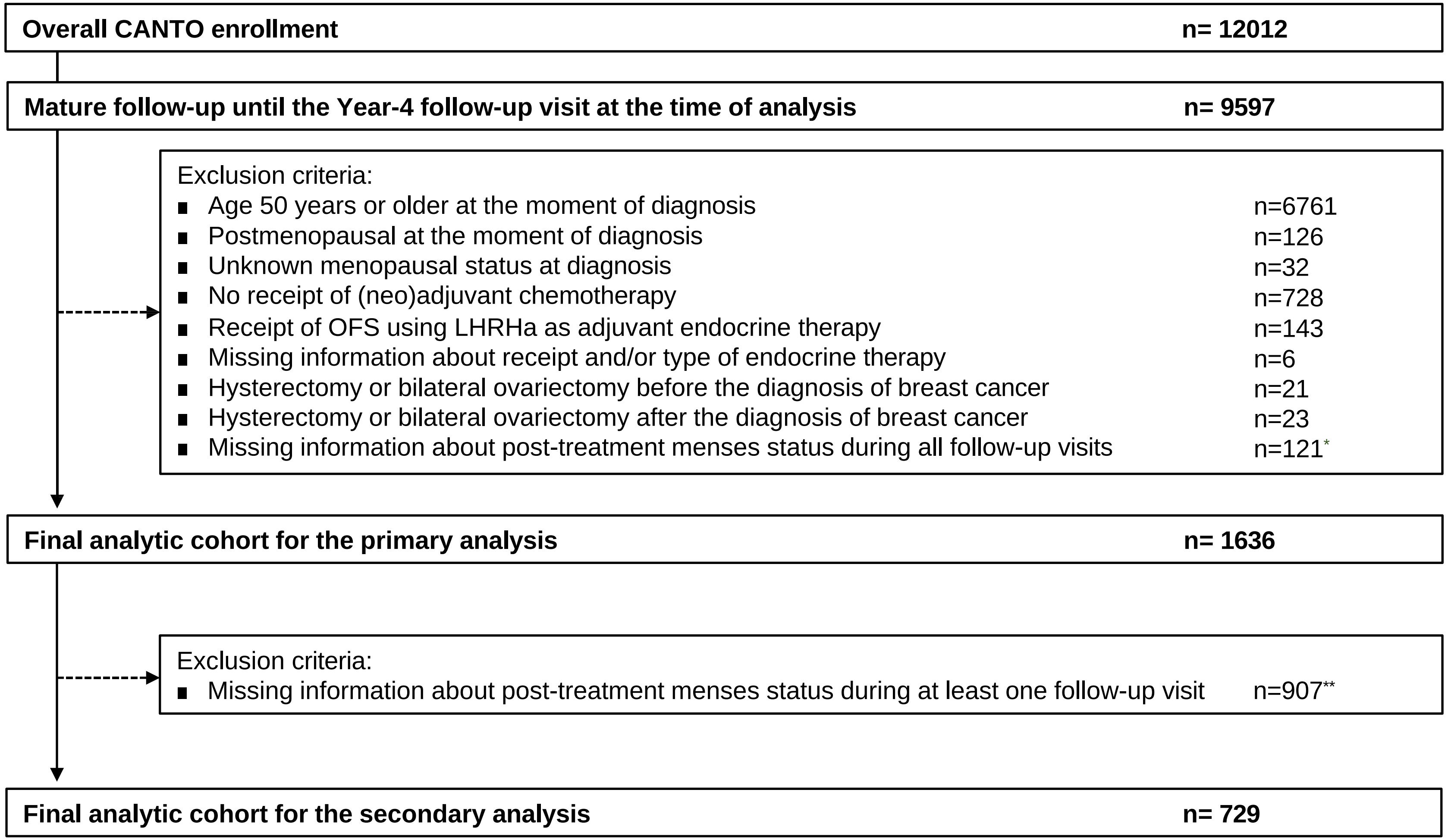

eFigure. Study Flowchart

© 2023 Kabirian R et al. *JAMA Network Open.*
