# Supplementary material for: Chemotherapy-Related Amenorrhea and Quality of Life Among Premenopausal Women With Breast Cancer
Source: JAMA Netw Open. 2023 Nov 16;6(11):e2343910. doi: 10.1001/jamanetworkopen.2023.43910 (PMC10654794; doi:10.1001/jamanetworkopen.2023.43910)
Supplement: Supplement 2. — Data Sharing Statement [file jamanetwopen-e2343910-s002.pdf]

Data Sharing Statement

Kabirian. Chemotherapy-Related Amenorrhea and Quality of Life Among Premenopausal Women With Breast Cancer. *JAMA Netw Open*. Published November 16, 2023. doi:10.1001/jamanetworkopen.2023.43910

Data
Data available: Data are available upon request to UNICANCER (https://www.unicancer.fr/en/)

Additional Information
Explanation for why data not available: Optional
